# The efficacy and safety of radiotherapy combined chemotherapy for laryngeal preservation in advanced laryngeal cancer: A protocol for systematic review and meta-analysis

**DOI:** 10.1097/MD.0000000000031899

**Published:** 2022-11-18

**Authors:** Lei Zhang, Jianfeng Li, Chenle Jia

**Affiliations:** a Department of Head and Neck Surgery Ward 1, Shanxi Province Cancer Hospital/Shanxi Hospital Affiliated to Cancer Hospital, Chinese Academy of Medical Sciences/Cancer Hospital Affiliated to Shanxi Medical University, Taiyuan, China; b Department of Otorhinolaryngology, Shanxi Provincial People’s Hospital Affiliated to Shanxi Medical University, Taiyuan, China

**Keywords:** advanced laryngeal cancer, chemotherapy, meta-analysis, overall survival, radiotherapy

## Abstract

The appropriate use of surgery or chemoradiotherapy-based approaches for organ preservation has been the subject of much debate. Unfortunately, there has been a lack of improvement in overall survival for patients with laryngeal carcinoma. In this study, we performed a protocol for systematic review and meta-analysis to evaluate the efficacy and safety radiotherapy combined chemotherapy for laryngeal preservation in advanced laryngeal cancer

**Methods:**

This protocol is reported in accordance with the Preferred Reporting Items for Systematic Review and Meta-Analysis Protocols (PRISMA-P) 2015 statement. We will search the PubMed, Cochrane Library, EMBASE, and Web of Science databases from the inception dates to October, 2022, using the keywords “laryngeal cancer,” “radiotherapy”, and “chemotherapy.” Cochrane “bias risk” tool is used to assess the bias risk of the quality of the included literature. All calculations were carried out with RevMan V.5.3 software.

**Results:**

The results of this study will provide evidence for judging whether radiotherapy combined chemotherapy is superior to surgery for treatment of advanced laryngeal cancer.

**Conclusion:**

This review will provide directions and recommendations for future research and clinical practices of radiotherapy combined chemotherapy for laryngeal preservation in advanced laryngeal cancer.

## 1. Introduction

Squamous cell carcinoma (SCC) of the larynx continues to be the commonest cancer of the head and neck in many Western countries.^[1,2]^ Major risk factors include smoking and alcohol consumption.^[3,4]^ Other risk factors include asbestos exposure, industrial pollution, history of larynx cancer in a first-degree relative, and inadequate intake of antioxidant micronutrients found in fresh fruit and vegetables.^[5,6]^ Males are more commonly affected, and most patients are aged over 40 years. While many countries have recently reported a decline in overall number of cases of larynx cancer,^[7]^ it would appear that this decrease is mainly due to the decreased number of cases affecting males, with a stable or increasing number of cases affecting females. These changes in epidemiology of larynx cancer have been attributed to changes in smoking patterns. The larynx has a key role in many essential functions, including speech production, swallowing, airway protection, and breathing.^[8]^ Disruption of any of these functions, by either the tumor or the treatment, may have devastating consequences for the patient. Therefore, besides achieving tumor control, the other major aim of laryngeal cancer treatment is to optimize functional outcomes.^[9,10]^ Although this is usually possible in early larynx cancers, preserving laryngeal function in the setting of advanced cancer while still offering the optimum oncological outcome can be a difficult challenge.

Based on a guideline by American Society of Clinical Oncology, a larynx-preserving approach is an appropriate treatment option for most patients with T3 or T4 laryngeal cancers without tumor invasion through cartilage into soft tissues.^[11]^ They recommend that concomitant radiation therapy and chemotherapy is the standard of care for patients with T3–T4 laryngeal cancer and that preservation surgery is limited to selected patients.^[12]^ However, at many institutions chemoradiotherapy is the treatment of choice for most T3 laryngeal cancer. The use of chemoradiotherapy is associated with a high incidence of acute toxicity and disruption in laryngeal function.^[13]^ In this study, we performed a protocol for systematic review and meta-analysis to evaluate the efficacy and safety radiotherapy combined chemotherapy for laryngeal preservation in advanced laryngeal cancer.

## 2. Methods

### 2.1. Study registration

This protocol is reported in accordance with the Preferred Reporting Items for Systematic Review and Meta-Analysis Protocols (PRISMA-P) 2015 statement^[14]^ and the Cochrane Handbook for Systematic Reviews of Interventions. The protocol for this review has been registered in the International Prospective Register of Systematic Reviews (registration number: CRD42022370084).

### 2.2. Ethics and dissemination

Because our study will not include animals or individuals, ethical approval will not be required. Once the results of the study are obtained, they will be published in conferences or peer-reviewed journals.

### 2.3. Inclusion criteria for study selection

#### 2.3..1. Type of studies.

Only randomized controlled trials (RCTs) are included in our studies. Other designs, such as in vivo, in vitro, case reports, retrospective studies, and non-RCTs will be excluded. There are no restrictions on languages.

#### 2.3..2. Type of participants.

We will include studies on patients that are diagnosed as advanced laryngeal cancer. The sex, age, and race are not limited.

#### 2.3..3. Type of interventions.

We will include the studies applying radiotherapy combined chemotherapy for laryngeal preservation as the sole intervention in the experimental group, while the control group received total laryngectomy followed by radiation.

#### 2.3..4. Type of outcome measurements.

Primary outcomes were the 2- to 5-year overall survival and disease-free survival. Secondary outcome were quality of life and adverse event.

### 2.4. Search strategy

We will search the PubMed, Cochrane Library, EMBASE, and Web of Science databases from the inception dates to October, 2022, using the keywords “laryngeal cancer,” “radiotherapy”, and “chemotherapy.” The search strategy in PubMed is shown in Table 1. In addition, the reference lists of previously published systematic reviews were manually examined for further pertinent studies.

**Table 1:** Search strategy for PubMed.

#1 radiotherapy [Title/Abstract]
#2 radiation therapy [Title/Abstract]
#3 brachy therapy [Title/Abstract]
#4 tomotherpy [Title/Abstract]
#5 #1 OR #2 OR #3 OR #4
#6 chemotherapy [Title/Abstract]
#7 drug therapy [Title/Abstract]
#8 #6 OR #7
#9 laryngeal cancer [Title/Abstract]
#10 laryngocarcinoma [Title/Abstract]
#11 laryngeal neoplasm [Title/Abstract]
#12 laryngeal squamous cell carcinoma [Title/Abstract]
#13 pharyngolaryngeal cancer [Title/Abstract]
#14 #9 OR #10 OR #11 OR #12 OR #13
#15 #5 AND #8 AND #14

### 2.5. Study selection

Two independent researchers screened the study titles and abstracts according to the inclusion criteria. The full text of the studies potentially meeting the eligibility criteria were retrieved for a more detailed read to make a final decision regarding inclusion.

### 2.6. Data extraction and management

**Figure 1.**
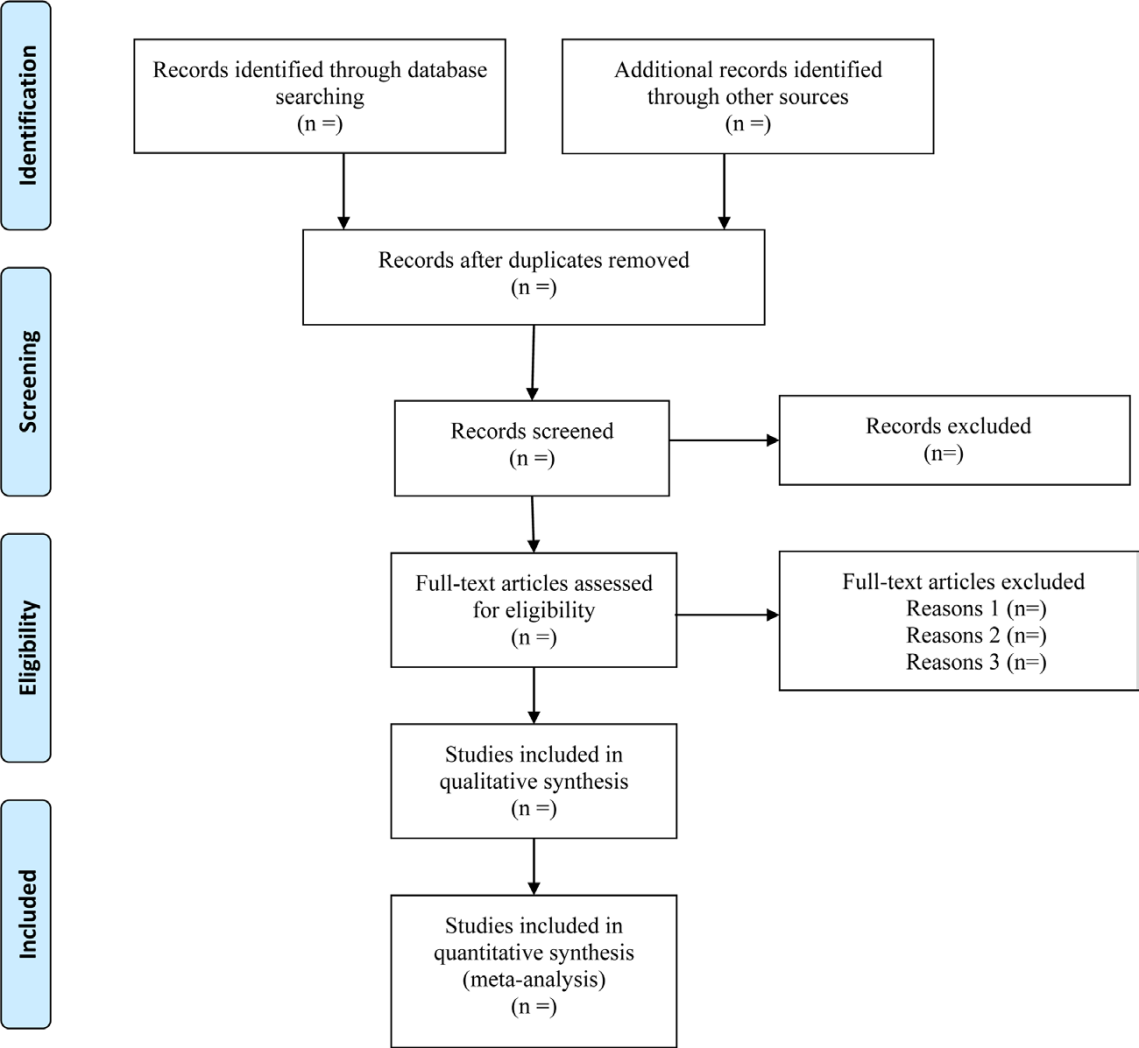
Flowchart of the study screening process.

The following data were extracted: lead author; publication year; country of origin; study design; sample size; age; tumor stage; outcome measures; and complications. Any differences of opinion will be resolved through group discussion or consultation with a third reviewer. When relevant data is not reported, we will contact the author via email or other means to obtain missing data. The Preferred Report items for the System Review and Meta-analysis flow diagram (Fig. 1) will be filled out after the screening study is completed to provide specific information.

### 2.7. Risk of bias

We will use the Cochrane “bias risk” tool to assess the bias risk of the quality of the included literature.^[15]^ Assessment items included random sequence generation, allocation concealment, blinding of participants and personnel, blinding of outcome assessment, whether the incomplete outcome data were adequately handled, evidence of selective outcome reporting, and other potential sources of bias.

### 2.8. Data synthesis

RevMan V.5.3 software will be used to analyze all data. In meta-analysis, Mantel–Haenszel method will be conducted to estimate the binary outcomes effect size, while inverse variance method will be conducted to estimate the continuous outcomes effect size. We will use the fixed-effect model to pool data whenever there is low heterogeneity. Analysis and treatment will be carried out first whenever there is high heterogeneity (*P* < .1 or *I*^2^ > 50%). If it cannot be solved, the random-effect model will be introduced to provide a more conservative effect estimation. For the research results with large heterogeneity that cannot be quantitatively integrated, a narrative report will be made. Sources of heterogeneity were assessed by sensitivity analysis, by excluding studies of low quality or small sample size, if the heterogeneity did not change significantly, the results were robust. Otherwise, the excluded studies may have been source of heterogeneity. In this study, fewer than 10 included studies were evaluated for publication bias using funnel plot, otherwise Egger regression test would be used.^[16]^

## 3. Discussion

Laryngeal preservation protocols, validated by the American Society of Clinical Oncology, are designed to preserve speech, breathing and swallowing functions of the larynx without altering survival.^[17]^ However, the long-term results of the first prospective study on this subject revealed a high rate (34%) of “unexplained” deaths after chemoradiation, raising the question of the long-term toxicity of these protocols. Analysis of the US National Cancer Data Base concerning laryngeal cancer, conducted by Hoffman et al in 2006,^[18]^ demonstrated a reduction of the 5-year survival in the group of patients treated with chemoradiation compared to the group treated by surgery. Patients with advanced laryngeal cancer are therefore faced with a therapeutic dilemma requiring a trade-off between preservation of laryngeal function offered by the laryngeal preservation protocol or the better survival provided by total laryngectomy.^[19]^ This classical “trade-off” situation does not only apply to advanced laryngeal cancer, but also concerns all fields of oncology. This study aims to evaluate the efficacy and safety of radiotherapy combined chemotherapy for laryngeal preservation in advanced laryngeal cancer. However, due to the limitations of the present review, more high-quality, multicenter RCTs are needed to further confirm the conclusion.

## Author contributions

**Writing:** Lei Zhang.

**Data analysis:** Jianfeng Li.

**Data collection and study design:** Chenle Jia.
